# A case series of Diffuse Glioneuronal Tumours with Oligodendroglioma‐like features and Nuclear Clusters (DGONC)

**DOI:** 10.1111/nan.12680

**Published:** 2021-01-12

**Authors:** Jessica C Pickles, Kshitij Mankad, Miren Aizpurua, Simon ML Paine, Leslie R Bridges, Fernando Carceller, Elwira Szychot, Mark Walker, Amy R Fairchild, Talisa Mistry, Olumide Ogunbiyi, Alice Rolland, Thomas J Stone, Carryl Dryden, Dilys Addy, Elisa Garimberti, Jane Chalker, Felix Sahm, David TW Jones, Darren Hargrave, Thomas S Jacques

**Affiliations:** ^1^ Developmental Biology and Cancer (DBC) Research & Teaching Department UCL Great Ormond Street Institute of Child Health London UK; ^2^ Department of Histopathology Great Ormond Street Hospital for Children NHS Foundation Trust London UK; ^3^ Department of Radiology Great Ormond Street Hospital for Children NHS Foundation Trust London UK; ^4^ Department of Clinical Neuropathology King’s College Hospital NHS Foundation Trust London UK; ^5^ Queen’s Medical Centre Campus Nottingham University Hospitals NHS Trust Nottingham UK; ^6^ Department of Neuropathology St George’s Hospital NHS Trust London UK; ^7^ Children and Young People’s Unit The Royal Marsden NHS Foundation Trust Surrey UK; ^8^ Cellular Pathology University Hospital Southampton NHS Foundation Trust Southampton UK; ^9^ Département de Neurochirurgie Centre Hospitalier Universitaire Montpellier France; ^10^ Specialist Integrated Haematology and Malignancy Diagnostic Service‐Acquired Genomics Great Ormond Street Hospital for Children NHS Foundation Trust London UK; ^11^ Hopp Children’s Cancer Center Heidelberg (KiTZ University Hospital Heidelberg Heidelberg Germany; ^12^ Department of Neuropathology University Hospital Heidelberg Heidelberg Germany; ^13^ Clinical Cooperation Unit Neuropathology German Consortium for Translational Cancer Research (DKTK German Cancer Research Center (DKFZ Heidelberg Germany; ^14^ Pediatric Glioma Research Group German Cancer Research Center (DKFZ Heidelberg Germany; ^15^ Department of Paediatric Oncology Great Ormond Street Hospital for Children NHS Foundation Trust London UK

**Keywords:** brain tumour, paediatric, DNA methylation classification, glioneuronal tumour, monosomy 14

## Abstract

In this study, we report three paediatric cases of Diffuse Glioneuronal Tumours with Oligodendroglioma‐like features and Nuclear Clusters (DGONC).

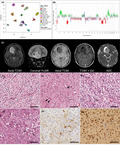

We read with interest Deng *et al*.’s recent manuscript concerning a newly molecularly defined glioneuronal CNS tumour entity, termed diffuse glioneuronal tumour with oligodendroglioma‐like features and nuclear clusters (DGONC).^1^ DGONCs were identified by a distinctive methylation profile that segregated away from previously recognised molecular groups of CNS tumours. On further review, the authors describe DGONCs as a novel glioneuronal tumour with key neuropathological features including oligodendroglioma‐like perinuclear halos and nuclear clusters. At a genetic level, their cohort of 31 DGONCs was found to have recurrent monosomy of chromosome 14 (97%), gain of 1q (26%) and 17q (58%), and loss of 19q (35%), but unlike well‐characterised glioneuronal tumours, no other defining genetic alterations were identified.

We reviewed our existing paediatric CNS tumour cohorts to determine if we could identify additional cases of DGONC. We reviewed 123 cases with an undetermined final diagnosis originating from two previously described cohorts with existing methylation array data.^2^ From these, we selected tumours that failed to classify using the most up‐to‐date version of the methylation classifier developed by the DKFZ^3^ (MNPv11b6) and that had monosomy of chromosome 14.^1^ We then performed unsupervised hierarchical clustering using the 10,000 most variably methylated probes for our candidate‐DGONC samples together with a cohort of 18 DGONC reported in the original article,^1^ along with tumours with a different confirmed molecular diagnosis.^2^, ^3^ Three samples reported were found to have methylation profiles with good similarity to Deng *et al*.’s published cases (Figure 1A, labelled GOSH DGONC) and one patient was later identified as having a second tumour biopsy taken a year later, which also clustered with the reference DGONC cohort.

**FIGURE 1:**
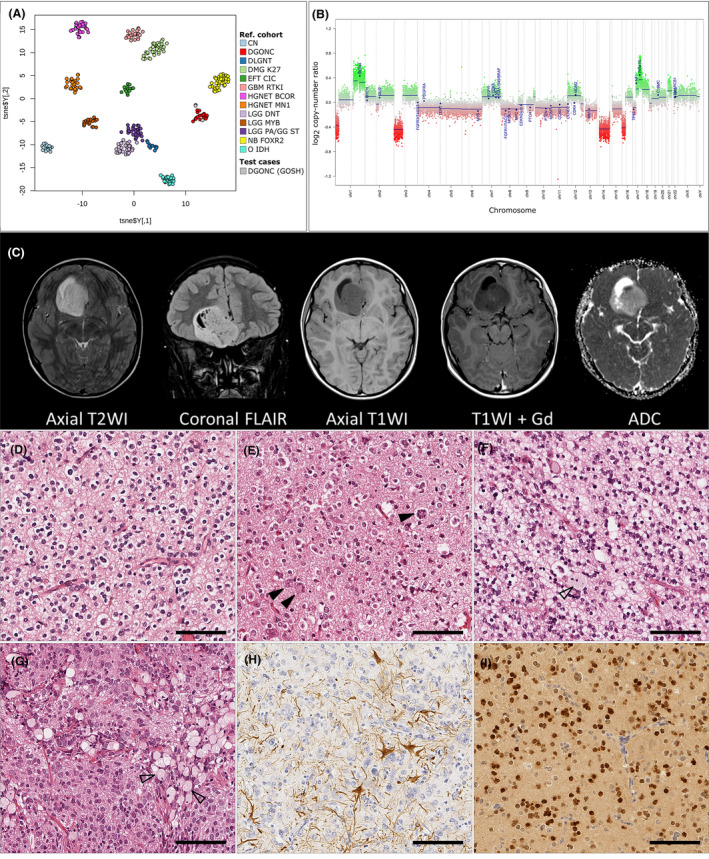
Summary of presenting features in three paediatric DGONC. (A) DNA methylation profiling of the new DGONC cases with existing reference cohorts available online via Heidelberg Molecular Neuropathology platform, https://www.molecularneuropathology.org/mnp ^3^ and those held locally with a confirmed molecular classification (calibrated score using MNPb11v6 >0.9).^2^ FS and DTWJ kindly provided reference DGONC samples.^1^ Samples are coloured according to their methylation class. t‐SNE parameters: perplexity = 20, theta = 0.5, dims = 2. (B) Representative copy number plot (DGONC_3) showing previously described features of DGONC: monosomy of chr14, gains in 1p and 17p. Additionally, we noted focal losses in 1p and 3p that were common in our cases series. (C) Radiological features (DGONC_3) of an 11‐year‐old boy presenting with a mass in the right medial frontal lobe, heterogeneously hyperintense on T2‐ and FLAIR‐weighted sequences with some internal cysts, poorly enhancing with contrast, and rather heterogeneous on diffusion, with a small focus of low ADC values. The CT scan (not shown) revealed calcification in the wall of the cystic area. (D–I) Representative histopathological features observed. Predominant features included rounded nuclei with perinuclear haloes (D–E), clear cell morphology, vascular proliferation and frequent areas of neutrophil (D–G). We observed nuclear aggregation (E, arrows), giant cells and large foamy cells (G). DGONC were negative for glial markers, for example, GFAP (H) and positive for neuronal and oligodendrocyte markers, for example, OLIG2 (I). See extended data for summary table of immunohistochemistry available and additional radiology. Abbreviations (A): CN: central neurocytoma, DGONC: Diffuse Glioneuronal tumour with Oligodendroglioma‐like features and Nuclear Clusters, DLGNT: diffuse leptomeningeal glioneuronal tumour, DMG K27: diffuse midline glioma with H3 K27M mutation; EFT CIC: Ewings‐like family tumour with CIC alteration; GBM RTKI: glioblastoma receptor tyrosine kinase I group; HGNET BCOR: high‐grade neuroepithelial tumour with BCOR alteration; HGNET MN1: high‐grade neuroepithelial tumour with MN1 alteration; LGG DNT: low‐grade glioma, dysembryoplastic neuroepithelial tumour; LGG MYB: low‐grade glioma MYB/MYB1; LGG PA/GG ST: low‐grade glioma, hemispheric pilocytic astrocytoma and ganglioglioma; NB FOXR2: neuroblastoma with FOXR2 alteration; O IDH: IDH glioma, 1q/19q codeleted oligodendroglioma; DGONC (GOSH): DGONC cases identified at our centre (DGONC_1 to DGONC_3, includes DGONC_1R)

All patients with a proposed DGONC were male children and, at the time of diagnosis, aged between 11 and 12 years. Case DGONC_1 had been diagnosed as a “malignant glioneuronal tumour”, while DGONC_2 and 3 had both been called “high‐grade neuroepithelial tumour”. These tumours presented as well‐defined cortical or subcortical supratentorial masses, and as previously described did not display any specific lobar predisposition. From the available radiology (Figure 1C and Figure S1), the tumours had no appreciable perilesional oedema, were hyperintense compared to cortex on T2 and FLAIR weighting and enhanced poorly with contrast if at all. They also demonstrated internal matrix calcification, with centrally low ADC values on diffusion‐weighted images. Imaging for the relapsed case indicated residual tumour but did not show additional radiological features.

All patients are alive and had relatively favourable outcomes, although follow‐up time following diagnosis varied between only 0.7 and 6.6 years. Following surgery, all patients received craniospinal radiation together with chemotherapy and are in complete remission. All patients received a gross total resection, the additional surgery for DGONC_1 was to remove persistent residual tumour following chemotherapy and there was no notable progression on radiology (Figure S1). One patient had a previous diagnosis and family history of Autistic Spectrum disorder and Hyperactivity, the other two patients had no known underlying conditions.

Morphologically all three cases had remarkably similar characteristics (see Figure 1D‐I and Table S1). They were diffusely arranged tumours composed of round cells with perinuclear haloes, akin to oligodendrogliomas or neurocytic tumours. There were nuclear clusters, at least some of which were part of multinucleate cells. Calcification, ganglion cells, apoptosis and foamy cells were commonly noted. Vascular proliferation was present in two of the four samples. The tumours were of moderate cellularity and the mitotic index ranged between 0.42 and 3.38 per mm^2^, with a focal maximum of 30% of cells staining positive for Ki67. The tumour cells did not express astrocytic markers (e.g. GFAP) but there was diffuse strong staining for neuronal markers Synaptophysin and NeuN, as well as the oligodendrocyte marker OLIG2. CD34 staining was present in the endothelium only. We did not observe histological differences between the primary and secondary DGONC_1 sample.

Apart from the methylation arrays, existing molecular workup did not identify recurrent molecular events (Table S1). Where data were available, no pathogenic variants were observed in the histone H3 genes, and no pathogenic variants or fusion events were detected in *BRAF*. Targeted panel sequencing on DNA and RNA was available for one case (a custom panel^4^ and Illumina TruSight RNA Pan‐Cancer fusion Panel, respectively) but failed to reveal any predicted driving molecular events. In addition to monosomy of chromosome 14, on review of the copy number plots, we noted a common focal loss of chromosome 1p (previously not reported) and loss of chromosome 3p.

In conclusion, we have independently identified three DGONCs with consistent histopathological, molecular and clinical features to those previously described. This study confirms that DGONC is a molecularly defined entity with a unique methylation profile and distinct histopathological features mimicking oligodendroglioma, with the presence of nuclear clusters. All three cases in our small series presented at a similar age (11–12 years) and are all male children, but we assume that this is coincidental as Deng *et al*.’s larger cohort did not observe a gender bias. Monosomy of chromosome 14 is the main recurrent molecular feature of DGONC, and there are no other currently described recurrent pathogenic genetic variants.

## DISCLOSURES

JCP, KM, MA, LRB, FC, ES, MW, ARF, TM, AR, TJS, CD, DA, EG, JC, FS and DH report that they have no conflicts of interest or disclosures. TSJ is the Editor in Chief and SMLP is a member of the editorial board of *Neuropathology and Applied Neurobiology*. The Editors of *Neuropathology and Applied Neurobiology* are committed to peer‐review integrity and upholding the highest standards of review. As such, this article was peer reviewed by independent, anonymous expert referees and the authors (including TSJ and SMLP) had no role in either the editorial decision or the handling of the paper. DTWJ has a patent pending: DNA methylation‐based method for classifying tumour species of the brain (EP3067432A1).

## AUTHOR’S CONTRIBUTIONS

JCP performed the analysis and wrote the manuscript. KM provided and interpreted radiological data, TSJ reviewed the histology. MA, SMLP, LRB, MK, DH and TSJ reported the original diagnostic and clinical findings. CD, DA, EG and JC reported the molecular findings. FC, ES, ARF, TM, OO and AR collected data and TJS provided bioinformatic support. FS and DTWJ provided DGONC reference data. All authors reviewed and accepted the final manuscript.

## ETHICAL APPROVAL

This study was covered under ethical approval granted by BRAIN UK tissue bank (REC: 14/SC/0098, references 16/007 and 17/007).

### Peer Review

The peer review history for this article is available at https://publons.com/publon/10.1111/nan.12680.

## Supporting information

Fig S1

Table S1

## Data Availability

The data that support the findings of this study are available from the corresponding author upon reasonable request. This article was peer‐reviewed by independent, anonymous expert referees, and the authors (including TSJ and SMLP) had no role in either the editorial decision or the handling of the paper.
