# Supplementary material for: A case series of Diffuse Glioneuronal Tumours with Oligodendroglioma‐like features and Nuclear Clusters (DGONC)
Source: Neuropathol Appl Neurobiol. 2021 Jan 12;47(3):464–7. doi: 10.1111/nan.12680 (PMC8048648; doi:10.1111/nan.12680)
Supplement: Supplementary file 1 — Fig S1 [file NAN-47-464-s002.pdf]

Supplementary Figure S1.

A

B

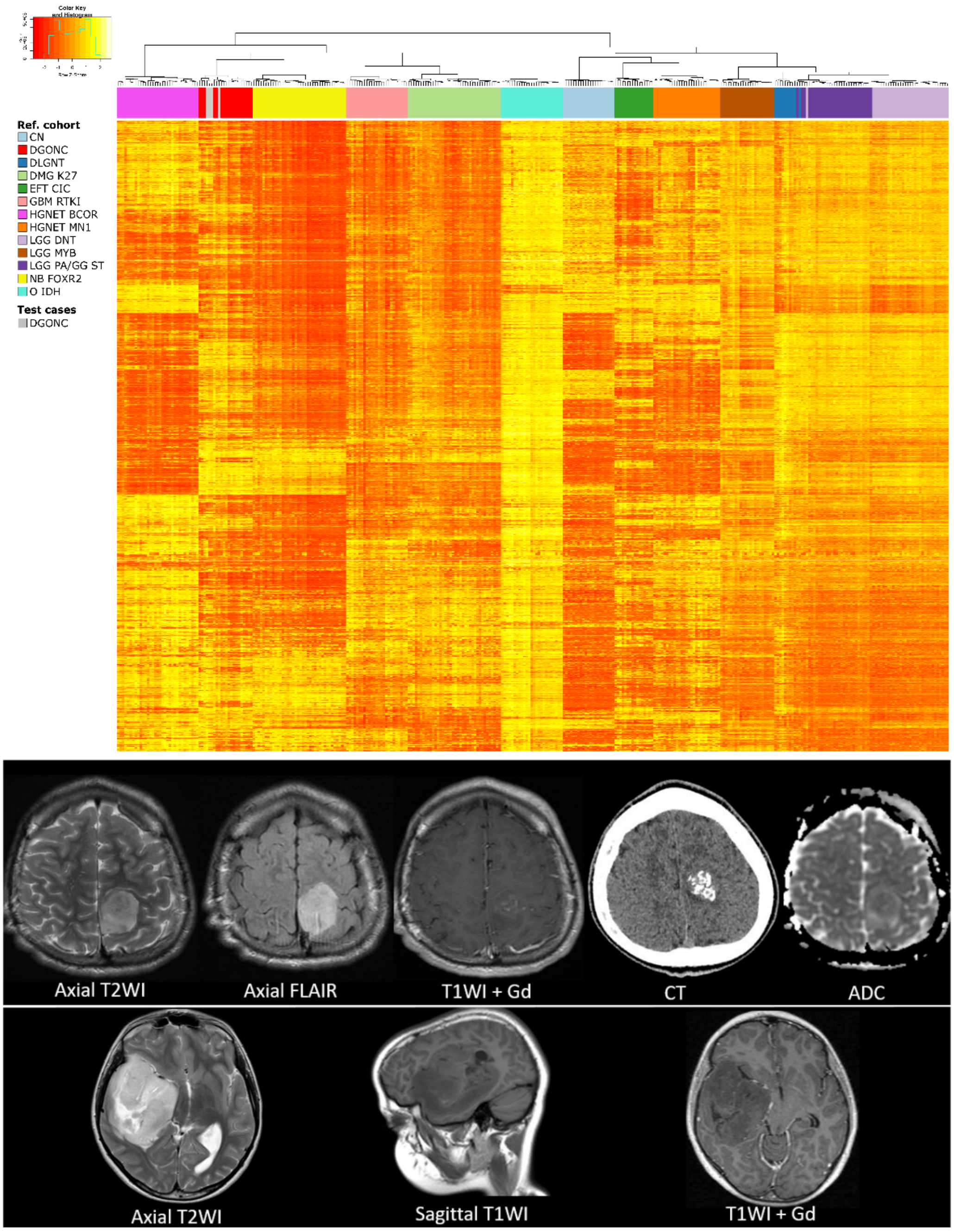

Supplementary Figure S1.

(A) DNA methylation profiling. Unsupervised hierarchical clustering of the top 10,000 most variably methylated probes, according to Euclidean distance between samples with Ward D2 linkage method. Each row represents a probe and each column a sample.

(B) Radiology available for the other two cases. Top row: A case presenting with a mass in the medial aspect of the left post central gyrus. It is homogenously hyperintense on T2 and FLAIR weighted sequences, enhances poorly with contrast, and demonstrates a densely calcified matrix (CT) and low ADC values on diffusion weighted imaging. Lower row: A case presenting with a large mass centred in the right temporal lobe, again hyperintense on T2 weighted sequence with internal cysts, and demonstrating very little enhancement with contrast. Other sequences and CT were not available on this case.
