# Supplementary material for: A case series of Diffuse Glioneuronal Tumours with Oligodendroglioma‐like features and Nuclear Clusters (DGONC)
Source: Neuropathol Appl Neurobiol. 2021 Jan 12;47(3):464–7. doi: 10.1111/nan.12680 (PMC8048648; doi:10.1111/nan.12680)
Supplement: Supplementary file 2 — Table S1 [file NAN-47-464-s001.pdf]

Supplementary Table S1.

Supplementary Table S1. Summary of (A) clinical, (B) morphological, (C) immunohistochemical and (D) molecular features in paediatric DGONC.

Ages are approximate. CSP-RT: craniospinal radiotherapy. CT: chemotherapy. HART: Hyperfractionated accelerated radiotherapy. NTR: near total resection. GTR: gross total resection. Mod: moderate. ('.') Indicates that a test was not performed.

Pathological review was performed by TSJ and cases were assessed for features occurring in previously described DGONC (1). % Ki-67 positive was determined in the region of highest labelling. Mitotic index was calculated as the number of mitotic counts per mm^2. In addition, we noted the presence of the following features: calcification, foamy cells, ganglion cells and apoptosis. Immunohistochemical staining was assessed by staining intensity (0 = negative; 1 = weak; 2 = moderate; 3 = strong); staining extent (+ <10% tumour cells positive; ++ 10%–50% positive; +++ >50% positive).

BRAF (exon 15) was assessed by HRM curve analysis, histone genes (exon 1) by Sanger sequencing, *BRAF* and *EWSR1* fusions were assessed by RT-PCR. Results from the DKFZ methylation classifier (MNPv11b6) and copy number plots were interpreted as previously described(2). A calibrated score ≥0.9 is necessary for reliable methylation class prediction (3). Custom NGS panels were performed as previously described(4) and using the Illumina TruSight RNA Pan-Cancer fusion Panel.

A. Clinical details							
**Mean age at diagnosis (min-max)**	**Gender ratio (M:F)**	**Mean survival from 1st diagnosis (min-max)**	**Last known status**	**Tumour location**	**Surgery**	**Treatment**	**Progression**
11<br>(11-12)	1:0	3.4<br>(0.7-6.6)	Alive	Right temporo-parietal (n=1);<br>Left fronto-parietal (n=1);<br>Frontal lobe, right medial basal (n=1)	1st NTR, 2nd GTR (n=1);<br>Biopsy, GTR (n=1);<br>GTR (n=1)	CSP-RT (two courses of HART),<br>CT (Milan protocol with high dose cyclophosphamide) (n=1)<br>Focal RT, 54Gy in 30# with concomitant CT (Temozolomide) followed by 12 cycles of adjuvant Temozolamide (n=1)<br>CSP-RT (St Judes/CCLG ET protocol),<br>CT (St Judes protocol) (n=1)	2nd surgery due to persistent residue post CT (n=1)<br>Complete remission (n=2)

Supplementary Table S1.

	B. Morphological features												
**Case**	**Cellularity**	**Clear cell features**	**Tumour cell morphology**	**Architectural pattern**	**Proliferation index**	**Mitotic counts per mm^2**	**Vascular proliferation**	**Neuropil-like islands**	**Nuclear clusters / Multinucleate cells**	**Calcification**	**Foamy cells**	**Ganglion cells**	**Apoptosis**	**Other morphology**
DGNOC_1	Mod.	Y	Oligodendrocyte-like	Diffuse	30%	2.95	N	N	Y	Y	Y	Y	Y	Areas of high cellularity with nuclear pleomorphism
DGNOC_1R	Mod.	Y	Oligodendrocyte-like	Diffuse	30%	3.38	Y	N	Y	N	Y	Y	N	Similar to primary tumour, with secondary changes from previous surgery
DGNOC_2	Mod. (variable)	Y	Oligodendrocyte-like	Diffuse	NA	2.11	Y	N	Y	Y	N	Y	Y	Some angular nuclei, mild to mod. nuclear pleomorphism
DGNOC_3	Mod.	Y	Oligodendrocyte-like	Diffuse	20%	0.42	N	Y	Y	Y	Y	Y	Y	Some fibrillary matrix, some myxoid

	**C. Immunohistochemical staining**							
**Case**	**GFAP**	**SYN**	**NEUN**	**OLIG2**	**CD34**	**S100**	**H3K27M**	**G34R**
DGNOC_1	0	2, +++	3, +++	3, +++	vascular only	2, ++	0	.
DGNOC_1R	0	3, +++	3, ++	3, +++	vascular only	2, ++	0	.
DGNOC_2	.	.	2, ++	3, +++	.	.	0	.
DGNOC_3	0	3, +++	.	3, +++	.	.	0	0

Supplementary Table S1.

	D. Molecular testing												
	Single gene analysis							Methylation array				NGS	
Case	*BRAF*	*H3F3A*	*HIST1H3B*	*HIST1H3C*	*IDH1/2*	BRAF fusion	EWRS1:ATF1 fusion	Methylation Class (c.s.)	Family Class (c.s)	MGMT prediction	Copy number plot	DNA custom gene panel	TruSight RNA PanCancer panel
DGNOC_1	WT	WT	WT	.	.	Not found	.	CNS NB, FOXR2 (0.3209)	N/A	unmeth	Chr loss: 1p (focal), 3p, 6, 11p, 14. Chr gain: 17q	.	.
DGNOC_1R	.	.	.	.	.	.	.	DMG, K27 (0.1460)	N/A	unmeth	Chr loss: 1p (focal), 3p, 6, 11p, 14. Chr gain: 17q	.	.
DGNOC_2	.	WT	WT	WT	WT	.	.	O IDH (0.1390)	MTGF IDH GLM (0.2015)	unmeth	Chr loss: 3, 7p, 10q, 11, 14, 19. Chr gain: 7q	.	.
DGNOC_3	.	.	.	.	.	.	Not Found	CNS NB, FOXR2 (0.6427)	N/A	unmeth	Chr loss: 1p (focal), 3p, 14, 16q. Chr gain: 1q, 17q	No variants, 17q gains	No variants
